# Interpretable machine learning-based clinical prediction model for predicting lymph node metastasis in patients with intrahepatic cholangiocarcinoma

**DOI:** 10.1186/s12876-024-03223-w

**Published:** 2024-04-19

**Authors:** Hui Xie, Tao Hong, Wencai Liu, Xiaodong Jia, Le Wang, Huan Zhang, Chan Xu, Xiaoke Zhang, Wen-Le Li, Quan Wang, Chengliang Yin, Xu Lv

**Affiliations:** 1 Department of General Surgery, Yan ’an People’s Hospital, Yan ’an, China; 2 Department of Cardiac Surgery, Fuwai Hospital Chinese Academy of Medical Sciences, Shenzhen, China; 3 Department of Orthopaedic Surgery, the First Affiliated Hospital of Nanchang University, Nanchang, China; 4 Senior Department of Oncology, Fifth Medical Center of PLA General Hospital, Beijing, China; 5 Department of thoracic surgery, the first affiliated hospital of Dalian Medical University, Dalian, China; 6 Graduate School of Shaanxi University of Chinese Medicine, Xianyang, 712046 China; 7 State Key Laboratory of MolecularVaccinology and Molecular Diagnostics & Center for Molecular Imaging and Translational Medicine, School of Public Health, Xiamen University, Xiamen, 361102 China; 8 Radiation Oncology Department, Fifth Medical Center of PLA General Hospital, Beijing, China; 9 Faculty of Medicine, Macau University of Science and Technology, Macau, China; 10 Department of General Surgery, Yixing Cancer Hospital, Yixing, Jiangsu 214200 China

**Keywords:** Intrahepatic cholangiocarcinoma, Machine learning algorithms, Lymph node metastasis, Web calculator

## Abstract

**Objective:**

Prediction of lymph node metastasis (LNM) for intrahepatic cholangiocarcinoma (ICC) is critical for the treatment regimen and prognosis. We aim to develop and validate machine learning (ML)-based predictive models for LNM in patients with ICC.

**Methods:**

A total of 345 patients with clinicopathological characteristics confirmed ICC from Jan 2007 to Jan 2019 were enrolled. The predictors of LNM were identified by the least absolute shrinkage and selection operator (LASSO) and logistic analysis. The selected variables were used for developing prediction models for LNM by six ML algorithms, including Logistic regression (LR), Gradient boosting machine (GBM), Extreme gradient boosting (XGB), Random Forest (RF), Decision tree (DT), Multilayer perceptron (MLP). We applied 10-fold cross validation as internal validation and calculated the average of the areas under the receiver operating characteristic (ROC) curve to measure the performance of all models. A feature selection approach was applied to identify importance of predictors in each model. The heat map was used to investigate the correlation of features. Finally, we established a web calculator using the best-performing model.

**Results:**

In multivariate logistic regression analysis, factors including alcoholic liver disease (ALD), smoking, boundary, diameter, and white blood cell (WBC) were identified as independent predictors for LNM in patients with ICC. In internal validation, the average values of AUC of six models ranged from 0.820 to 0.908. The XGB model was identified as the best model, the average AUC was 0.908. Finally, we established a web calculator by XGB model, which was useful for clinicians to calculate the likelihood of LNM.

**Conclusion:**

The proposed ML-based predicted models had a good performance to predict LNM of patients with ICC. XGB performed best. A web calculator based on the ML algorithm showed promise in assisting clinicians to predict LNM and developed individualized medical plans.

Intrahepatic cholangiocarcinoma (ICC) is the second most common pathological type of primary liver cancer, after hepatocellular carcinoma (HCC) [1], accounting for approximately 10%~20% of all cases [2, 3]. The incidence rate of ICC has increased during the last several decades [1, 4, 5]. ICC has an extremely poor prognosis and also is a highly invasive malignant tumor [1, 2], the 5-year overall survival rate has been reported in the range of 22–44% [1, 6]. In the progress of invasion, lymph node metastasis (LNM) is commonly observed, the rate of lymph node metastasis is about 25%~50% [7]. Median survival times in ICC patients with no lymph node metastasis is 19.0~37.6 months, whereas those with LNM had only 9.0~22.9 months [8]. Surgery serves as the major method of treatment for ICC patients [3], lymphadenectomy is crucial to accurately stage the disease and guide decisions around adjuvant chemotherapy [9]. However, no international consensus has been reached on management of the lymph nodes during the operation. Based on the essential impact of lymph node metastasis on staging and treatment in ICC patients, the identification of the probability of LNM has great effective clinical significance [10, 11].

Usually, radiological image is a main method to judge lymph node status, however the limitations can’t be ignored. The sensitivity and specificity of CT diagnosis is 40%~50% and 77%~77%, respectively, and MRI is lower than CT scan [12], although the positron emission tomography (PET/CT) has higher accuracy in the assessment of LNM in patients with ICC [13], due to the high cost of PET/CT, it is not possible to routinely monitor all patients with this method. In clinic practice, pathology serves as the gold standard for LNM, but detailed information is unknown until after surgery [10]. Thus, reliable prediction models of LNM through clinical factors are urgent required. Various prediction models [3, 7, 14–18] have been constructed to predict the prognostic of ICC patients. As for the prediction model of LNM, although previous studies [7–9, 16, 18–20] have integrated potential risk factors to construct several predictive models, we don’t found that current studies have developed and validated a model to predict LNM using ML algorithms.

Recently, Machine learning (ML) algorithm, as an emerging and popular type of artificial intelligence (AI), has attracted more and more attention due to the ability to predict events occurrence and outcome and was widely applied to health-care data analysis, aid in clinical decision-making [21], especially in predicting possibility of metastatic diseases in malignant tumor patients [22, 23].

Herein, we developed and validated ML-based models using clinical characteristics to predict the probability of LNM in ICC patients. And a machine learning algorithm with the strongest predictive power is visualized by using a web calculator. This study will be helpful for surgical planning and clinical management.

## Methods

### Patient population

The Ethics Commission of the Fifth Medical Center of PLA General Hospital approved this present retrospective study (2019002D). All patients signed informed consent before surgery. Between Jan 2007 and Jan 2019, 345 patients who underwent surgical resection and regional lymphadenectomy for ICC at the Fifth Medical Center of PLA General Hospital were enrolled in this study.

Included patients had ICC proven by histopathology. The exclusion criteria were as follows:(1) history of other malignant tumors; (2) undergoing anticancer therapy (radiotherapy or chemotherapy) for liver malignancy before surgery; (3) primary liver cancer with mixed types and metastatic liver tumors; (4) incomplete clinical records.

### Feature selection for modeling

The collected clinical features were conducted dimension reduction and screened by LASSO analysis, which was utilized to select optimal features with non-zero coefficients as risk factors from the development cohort and minimize the risk of overfitting [24]. The results of backward step-wise regression analysis in the optimal features datasets were included in univariate and multivariate logistics regression analyses. Then, the clinical variables in the univariate regression independently related to LNM were further analyzed by multivariate regression analysis, the LNM independently related variables with *p*-values < 0.05 in multivariate regression analysis were presented to generate predictive models for patients with ICC.

### Development of the predictive models

Machine learning algorithms outperform traditional regression methods when predicting the outcomes [25]. In this study, we implemented six ML algorithms to develop predictive models as follows: Random Forest (RF), Logistic regression (LR), Extreme gradient boosting (XGB), Gradient boosting machine (GBM), Multilayer perceptron (MLP), and Decision tree (DT) [26, 27]. Afterward we employed 10-fold cross-validation in the model development and calculated the average value of AUC of the receiver operating characteristic curve to compare prediction power of illustrated models. Using the Permutation Importance analysis to assess the importance of predictors in each ML-based model predicting LNM. We calculated Pearson’s correlation coefficients to assess collinearity among the variables and plotted the correlation heat map. Finally, based on the best-performing model, we designed a web calculator as a predictive tool easily and accurately accessible to clinicians, making it possible to quantitatively calculate the individual probability of LNM.

### Statistical analysis

We applied the mean ± standard deviation (SD) to described the continuous variables and compared using the student’s t tests, while categorical variables were expressed as percentages or frequencies and determined the significant difference using the chi-square test. Statistical analysis was performed with R software (version 4.05), including logistics regression analysis, baseline tables. Machine learning models and web calculator were built using Python (version 3.8). Statistical significance levels were set at .05.

## Results

### Baseline characteristics

The baseline characteristics between ICC patients with LNM and without LNM are detailed shown in Table 1. According to the inclusion and exclusion criteria, a total of 345 ICC patients have been enrolled. The median survival time was 20.49 months in patients without LNM, which was significantly different from patients with LNM (the median survival time = 7.83 months). Patients with LNM had higher mortality and shorter survival time than those without LNM (*p* < 0.001). This revealed that lymph node metastasis has a huge negative effect on survival of ICC patients. Patients with tumor diameter > 5 cm were more susceptible to metastases in lymph node. In addition, smoking, ALD (alcohol liver disease), white blood cell (WBC), boundary and diameter were all significantly associated with LNM (*P*-value < 0.05). However, there were no significant differences in NAFLD (non-alcoholic fatty liver disease), hyperlipidemia, image number, and Mg between the two groups (Table 1, *p* > 0.05).

**Table1:** Baseline data of lymphatic metastasis in patients with intrahepatic cholangiocarcinoma

Characteristics	level	No(*N*=117)	Yes(*N*=228)	*p*
Status (%)	alive	48 (41.0)	27 (11.8)	<0.001
	dead	69 (59.0)	201 (88.2)	
Times (mean (SD))	NA	20.49 (19.05)	7.83 (5.88)	<0.001
NAFLD (%)	no	114 (97.4)	217 (95.2)	0.472
	yes	3 (2.6)	11 (4.8)	
ALD (%)	no	82 (70.1)	194 (85.1)	0.002
	yes	35 (29.9)	34 (14.9)	
Smoking (%)	no	74 (63.2)	76 (33.3)	<0.001
	yes	43 (36.8)	152 (66.7)	
Hyperlipidemia (%)	no	113 (96.6)	226 (99.1)	0.202
	yes	4 (3.4)	2 (0.9)	
Image number (%)	double	29 (24.8)	50 (21.9)	0.293
	more	5 (4.3)	4 (1.8)	
	single	83 (70.9)	174 (76.3)	
Boundary (%)	no	56 (47.9)	195 (85.5)	<0.001
	yes	61 (52.1)	33 (14.5)	
Diameter G (%)	<5cm	54 (46.2)	23 (10.1)	<0.001
	>10cm	7 (6.0)	29 (12.7)	
	5-10cm	56 (47.9)	176 (77.2)	
WBC (mean (SD))	NA	6.05 (2.40)	7.18 (1.96)	<0.001
Mg (mean (SD))	NA	0.87 (0.07)	0.90 (0.29)	0.269

### LASSO and logistic regression for models development feature selection

**Fig. 1:**
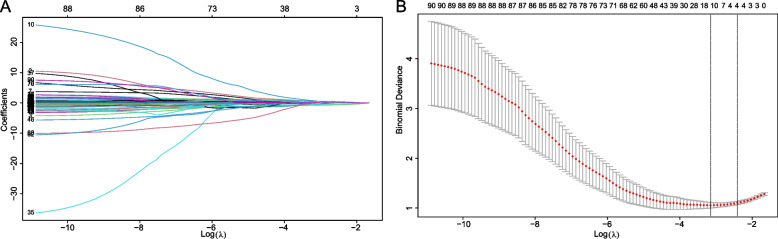
The results of LASSO regression

Of all clinical features, 93 features were reduced to 12 potential predictors with nonzero coefficients in the LASSO logistic regression analysis (Fig. 1). LASSO analysis, a method suitable for data dimension reduction and feature selection of high-dimensional data, makes the relatively unimportant features coefficients zero by the regularization technique [28]. By backward stepwise regression, we selected 9 variables to univariate and multivariable logistics regression. The univariate logistics regression analysis found that 5 factors related to LNM, then according to the results in multivariable logistics regression analysis, ALD (yes, OR = 0.25, 95% CI = 0.13-0.5, *p*<0.001), smoking (yes, OR = 3.83, 95% CI =2.13-6.88, *p*<0.001), boundary (yes, OR = 0.31, 95% CI = 0.17-0.55, *p*<0.001), Diameter (5–10 cm, OR = 3.14, 95% CI = 1.63-6.06, *p* = 0.000; >10 cm, OR = 5.89, 95% CI = 2.06–16.85, *p* = 0.001), and WBC (the serum level>7180/µL, OR = 1.18, 95% CI = 1.03-1.35, *p* =0.016) were identified as independent factors associated with LNM in patients with ICC (Table 2), among five variables, a distinct boundary and ALD were independent protective factors. Therefore, machine learning models were developed based on above five independent predictive factors (Table 2).

**Table2:** Univariate and multivariable logistics regression

Characteristics	OR	CI	*P*	OR	CI	*P*
ALD						
no	Ref	Ref	Ref	Ref	Ref	Ref
yes	0.41	0.24-0.7	0.001	0.25	0.13-0.5	<0.001
Boundary						
no	Ref	Ref	Ref	Ref	Ref	Ref
yes	0.16	0.09-0.26	<0.001	0.31	0.17-0.55	<0.001
Diameter G						
<5cm	Ref	Ref	Ref	Ref	Ref	Ref
>10cm	9.73	3.73-25.37	<0.001	5.89	2.06-16.85	0.001
5-10cm	7.38	4.16-13.09	<0.001	3.14	1.63-6.06	0.001
Hyperlipidemia						
no	Ref	Ref	Ref	Ref	Ref	Ref
yes	0.25	0.05-1.39	0.113	NA	NA	NA
Image number						
double	Ref	Ref	Ref	Ref	Ref	Ref
more	0.46	0.12-1.87	0.28	NA	NA	NA
single	1.22	0.72-2.06	0.467	NA	NA	NA
Mg	26.77	0.55-1299.91	0.097	NA	NA	NA
NAFLD						
no	Ref	Ref	Ref	Ref	Ref	Ref
yes	1.93	0.53-7.04	0.322	NA	NA	NA
Smoking						
no	Ref	Ref	Ref	Ref	Ref	Ref
yes	3.44	2.16-5.48	<0.001	3.83	2.13-6.88	<0.001
WBC	1.34	1.18-1.53	<0.001	1.18	1.03-1.35	0.016

### Performance of developed models

**Fig. 2:**
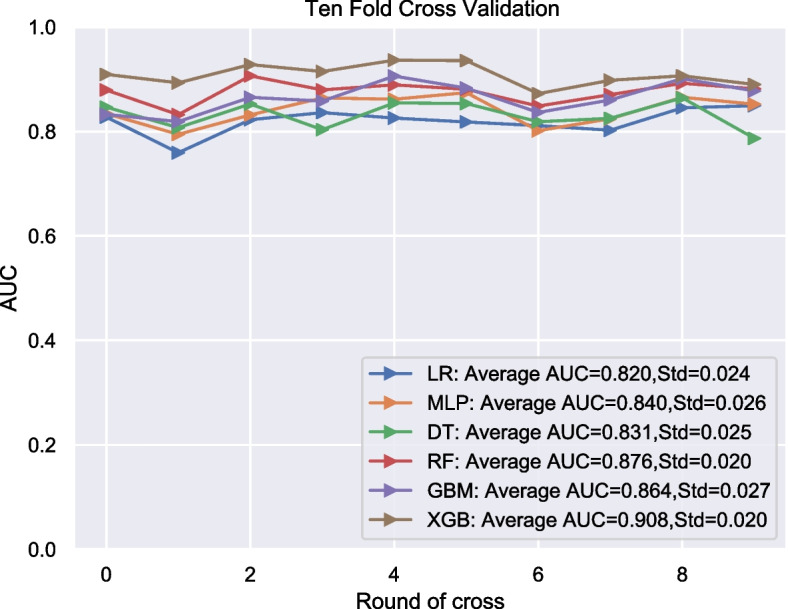
10-fold cross validation of machine learning algorithms

Six machine learning algorithm models based on the five variables were constructed. Internal validation was assessed, the performance of each machine learning algorithm was evaluated by 10-fold cross-validation, the average AUC values for evaluate performance in each model were calculated as follows: XGB: Average AUC=0.908; LR: Average AUC=0.820; MLP: Average AUC=0.840; DT: Average AUC=0.831; RF: Average AUC=0.876; GBM: Average AUC=0.864. As illustrated in Fig. 2. XGB algorithm had better accuracy in predicting LNM than the other five models.

### Variable importance and Pearson correlation of variables

**Fig. 3:**
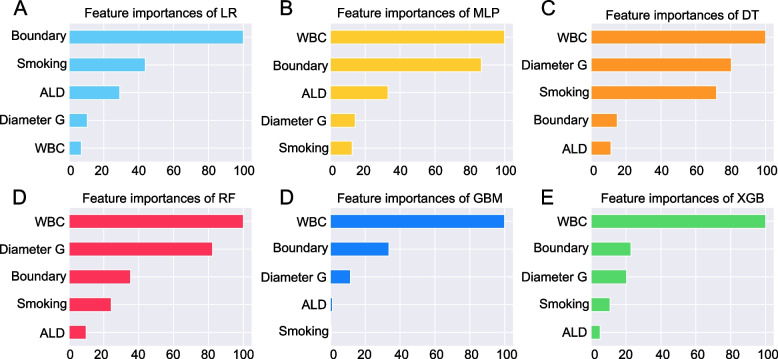
Importance of the independent variables

**Fig. 4:**
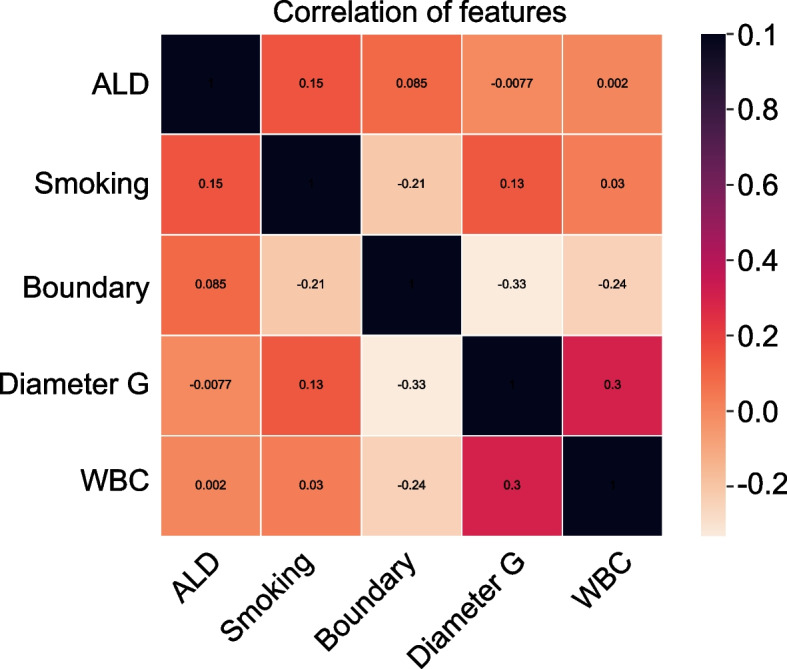
Correlations between the independent variables

Permutational importance quantified the variable importance in each ML algorithms (Fig. 3), WBC ranked first in five algorithms, the importance of variables in the XGB model is arranged in the following order: WBC, boundary, diameter G, smoking, ALD. In Fig. 4, we evaluated the correlation of the variables using Pearson’s correlation, and visualized the relationship of them via a heat map, indicating that no significant correlation and no collinearity among the variables for LNM, indicating that the variables are independent of each other and no collinearity among the variables. WBC, followed by boundary, were the most important features in XGB, a significant negative correlation had been found between them.

### Establishment of a web calculator

**Fig. 5:**
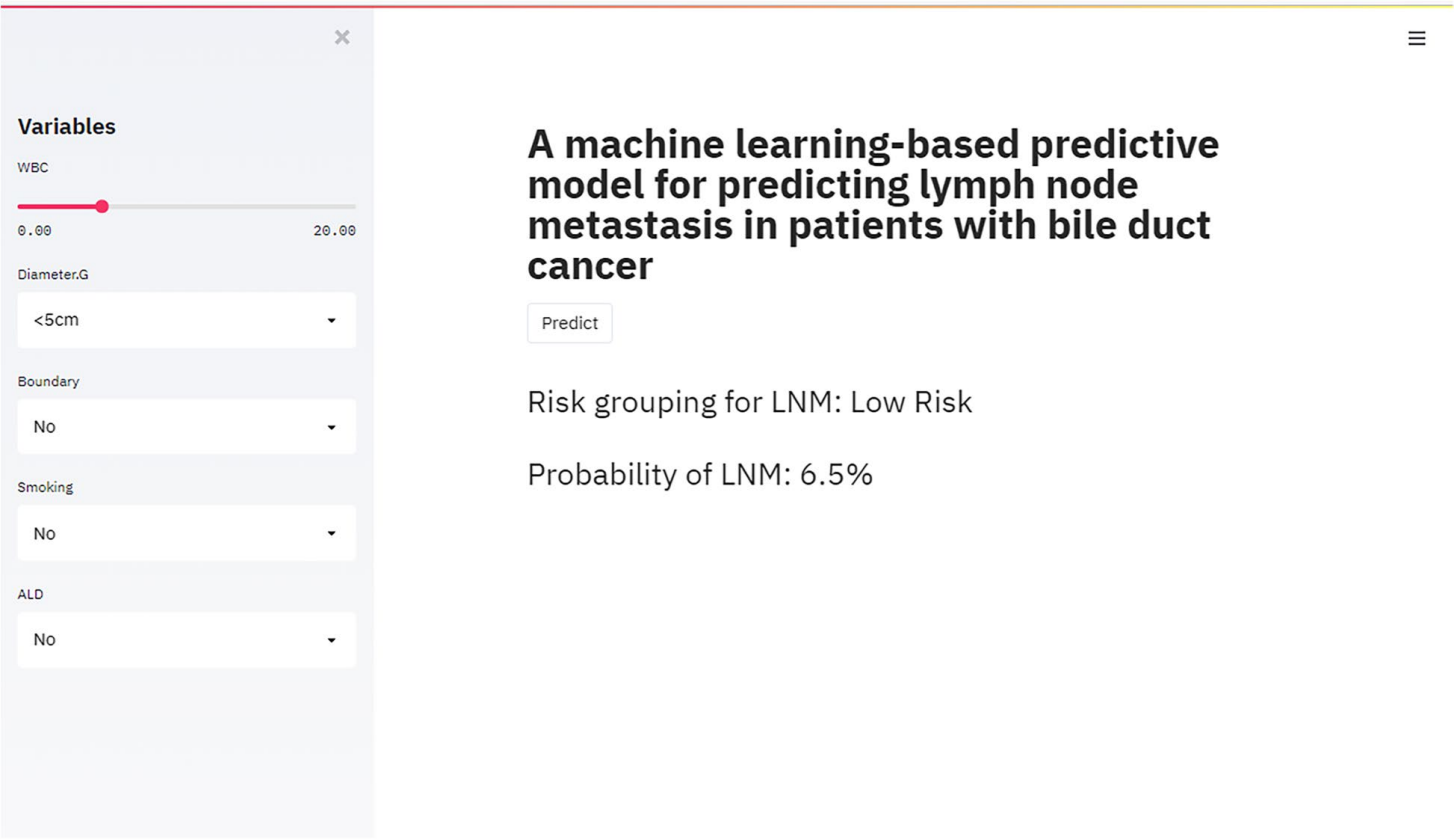
The web-based calculator for predicting LNM in patients with ICC

Based on the XGB model, we built an easy-to-use web calculator based on the XGB algorithm for clinicians to calculate the individualized likelihood of LNM in ICC patients with a simple input of easily accessible clinical variables (Fig. 5).

## Discussion

Intrahepatic cholangiocarcinoma originates from the malignant transformation of the bile ducts epithelium, and represents more aggressive compared to HCC [1], with the 5-year overall survival ranging from 15% to 40% [1, 6]. The incidence of LNM in ICC is much higher than that in HCC [29].Indeed lymph node status is critical for therapy selection and has been identified as one of the most important factors for prognosis [6]. A few of studies demonstrated that lymphadenectomy (LND) improved long-term survival outcome of ICC patients [30, 31], thus, LND should be a routine method for radical resection in ICC [32, 33]. Whereas other studies reported that LND didn’t improve survival outcome of ICC patients , with associated surgery-related complications [34, 35]. It’s reported that approximately 50% of the patients did not dissect lymph node dissection [36], which may result in mis-or under-staging and further compromised their outcomes [32, 36]. For ICC patients, accurate prediction of LNM will facilitate clinical treatment decision-making for the appropriate diagnosis and surgical planning.

Accordingly, we used a novel type of AI-machine learning-to predict LNM in ICC patients. Using ML algorithms, we developed and validated six models to predict LNM in 345 patients with ICC. We found that XGB model (average AUC=0.908) had greatest predictive performance in internal validation. Unlike some nomogram models [14, 19], we further provided dynamic construction. Consequently, based on the XGB model, a web calculator has been established to estimate visually individual probability of LNM and improved the applicability of the model.

In our study, multivariate logistic regression analysis founded that ALD, smoking, boundary, diameter, and WBC were independent predictive factors of LNM in patients with ICC (Table 2). As an independent risk factor, the influence of WBC on prognosis has been reported. Shirono et al [37] found that the serum WBC level was negatively associated with survival time in ICC patients, furthermore illustrated that patients with the WBC level was more than 6800/µL had a short survival time. In this study, we demonstrated that WBC was an independent predictor for the presentation of LNM in ICC patients. We also revealed that the risk of LNM was significantly increased when serum WBC level was more than 7180/µL. According to the permutation importance of variables in Fig. 3, WBC ranks first among the five prediction models and deserves the most attention when predicting LNM. WBCs include monocytes, lymphocytes and neutrophils. Monocytes have roles in promoting tumor invasion and angiogenesis [38]. In addition, tumor-associated macrophages developed from monocytes, can promote tumor lymphangiogenesis by the secretion of pro-lymphangiogenic factors and trans-differentiation into lymphatic endothelial cells [39]. Subimerb et al. reported that the monocyte in patients with Cholangiocarcinoma is correlated with a poor prognosis [40]. On the other hand, lymphocytes play an essential role in immune response, low counts may result in an insufficient immunological reaction against tumor progression and metastasis [38]. Previous research has revealed that lymphocyte to monocyte ratio (LMR) was associated with N stage and distant metastasis [41]. Peng et al. reported that the pre-LMR served as a predictor for early recurrence of Cholangiocarcinoma [42]. Meanwhile, a high neutrophil count was associated with poor prognosis and recurrence in ICC [43]. Stefan et al. reported that neutrophil to lymphocyte ratio was independently associated with worse overall survival among ICC patients [44]. In the present study, a high WBC level maybe reflect increasing in monocytes or neutrophil. The effects of monocytes, lymphocytes and neutrophils on lymph node metastasis should be further studied.

In addition, we concluded that tumors with diameter less than 5cm were less likely to occur LNM, which is similar to previous conclusion [20]. What’s more, we performed more detailed studies for tumor (diameter>5cm), according to multivariate logistics regression analysis results, compared to tumor with 5-10cm, larger tumor (diameter more than 10cm) had a higher metastatic risk to lymph nodes (OR:5.89 VS 3.14). Due to the biological growth behavior of ICC, larger tumor volume means that the tumor has a longer growth cycle and further increases the possibility of lymph node invasive risk.

In addition, the present study found that the type of ICC boundary on radiological image was closely related to LNM, a distinct boundary played a protective role in reducing the likelihood of LNM occurrence, similar result has been reported previously [20]. Microinvasion may reveal a possible mechanism of tumor aggressiveness to lymph nodules [45]. As showed in Fig. 4, boundary served as the second important feature after WBC. Two other independent predictive factors were ALD and smoking. A meta-analysis of eight studies [46] reported that alcohol was major risk factors for ICC. Drinking alcohol causes alcoholic liver disease, which is greatly associated with increased ICC risk [47], as smoking dose [48]. Nonetheless, the relationship between ALD, smoking and LNM in ICC patients was comprehended poorly. Interestingly, we found that ALD was a protective factor for LNM. This finding seems to contradict the existing literature identifying ALD as a risk factor for various cancers, including ICC [46, 47]. To reconcile this apparent paradox, we propose several hypotheses. First, ALD-induced immunosuppression may alter the host’s immune landscape, reducing the attack of immune cells on cancer cells and thus reducing the spread of lymphoid tumors (Gao & Bataller, 2011). Second, liver pathology associated with ALD, particularly cirrhosis, may adversely alter the hepatic microenvironment, impeding tumor cell migration and invasion due to tissue reorganization and vascular changes [49] . Third, there may be a potential selection for survival bias, whereby ALD patients who die prematurely due to liver disease complications do not have sufficient time to develop LNM, leading to an underestimation of the risk factors associated with LNM in long-lived populations. Finally, the chronic inflammatory state associated with ALD may inhibit tumor spread, contrary to the generally accepted view that inflammation promotes cancer progression [50, 51]. These considerations highlight the complexity and individual variability of tumor biology and underscore the need for further research to elucidate the mechanisms by which ALD affects ICC metastatic behavior, thereby providing new insights into therapeutic approaches and patient management. Smoking was significantly associated with LNM and was an independent risk factor for LNM. Therefore, in people with a preliminary diagnosis of ICC, we recommend smoking cessation. However, whether quitting smoking can reduce the risk of LNM in patients with a history of smoking needs to be further verified.

To our knowledge, this paper is the first study to develop and validate a predictive models for predicting LNM in ICC applying machine learning algorithms. The model distinguishes from linear models adopted by previous studies, which can maximize clinical parameters and improve the diagnosis accuracy.

The XGB model initially proposed by Chen et al. in 2016 possessed the best prediction performance [22], it has a high accuracy and fast processing time and has been regarded as a more reliable algorithm when the sample size is limited [52]. Therefore, XGB is suitable for our study which is a small sample from a single medical center.

Finally, we established a concise, visualizable and dynamic online application based on XGB model, the real-time risk of LNM can be calculated and more rational and specific treatment regimens for patients can be tailored according to the personal information. For example, when an ICC patient presented with the following clinical characteristics: tumor diameter less than 5 cm, no boundary, no smoking, ALD and serum WBC count is 5000/µL. We inputted above data into the web calculator, then the application integrated each factor and calculated automatically total probability of LNM, the output result was approximately 6.5% (Fig. 5), indicating that the patient had a low risk to lymph node metastasis. Therefore, we do not recommend further PET/CT monitoring and lymph node dissection.

## Conclusions

To sum up, we constructed a machine learning-based predictive model with a good performance to predict LNM in patient with ICC based on independent factors, including ALD, smoking, boundary, tumor diameter and WBC level. In addition, we did an attempt to translate research outputs into clinical practices by builting an online calculator, and the real-time predictive tool may aid in decision-making and management of ICC patients.

### Limitations

Some limitations in our study can’t be ignored. Firstly, as a retrospective study, selection bias was inevitable. In addition, the present study is small sample size from a single institution, our study is the lack of validation in an external dataset. In the future, external validation and large-scale multicenter studies will be required to validate our results. Thirdly, the inclusion of variables may affect the accuracy of the prediction model due to the highly subjective of the discrimination of tumor boundary and measure of diameter. Finally, there is a lack of analysis of LNM by neutrophils, lymphocytes and monocytes. According to previous studies [41], preoperative lymphocyte/monocyte ratio is associated with metastasis, and studying the subsets of WBC may improve the accuracy of prediction.

## Data Availability

The data used in this study can be obtained from the corresponding author on reasonable grounds.
